# Design and fabrication of a Wilkinson power divider with harmonic suppression for LTE and GSM applications

**DOI:** 10.1038/s41598-023-31019-7

**Published:** 2023-03-14

**Authors:** Gholamhosein Moloudian, Sepehr Soltani, Sirous Bahrami, John L. Buckley, Brendan O’Flynn, Ali Lalbakhsh

**Affiliations:** 1 Tyndall National Institute, University College Cork, Cork, T12R5CP Ireland; 2 School of Electrical Engineering, Shiraz University, Shiraz, Iran; 3 Department of Electrical Engineering, Pohang University of Science and Technology, Pohang, South Korea; 4 Macquarie University College, Macquarie University, Sydney, NSW 2109 Australia

**Keywords:** Electrical and electronic engineering, Electronics, photonics and device physics

## Abstract

Conventional Wilkinson power dividers (WPDs) can provide acceptable performance close to the nominal center frequency. However, these WPDs can also exhibit poor out-of-band performance while requiring a large footprint. In order to improve on the current state of the art, a modified microstrip WPD is proposed that exhibits a substantially improved stopband and high isolation. A lowpass filter (LPF) structure is utilized in both branches of the power divider to provide harmonic suppression. According to the obtained results, the input return loss (|*S*_11_|), output return loss (|*S*_22_|), output insertion loss (|*S*_21_|) and isolation (|*S*_32_|) are better than 34.2 dB, 26.2 dB, 3.52 dB and 31.2 dB, respectively. The proposed modified WPD has a wide 20 dB stopband (from 2.54 GHz to 13.48 GHz) and filters the second to seventh harmonics with attenuation levels of greater than 20 dB. The proposed WPD has a small size of 33.8 mm × 27 mm (0.42 *λ*g × 0.33 *λ*g), where *λ*g is the guided wavelength at the operating frequency of 1.8 GHz. The WPD has been fabricated and tested and shows good agreement between simulated and measured results and the proposed design has desirable characteristics for LTE and GSM applications.

## Introduction

In recent years, modern wireless communication systems have become increasingly important, and the demand for high-performance active and passive RF/Microwave components such as antennas^1–3^, filters^4–15^, power dividers^16–32^, mixers^33^ and multiplexers^34–38^ has grown substantially. In particular, passive microstrip devices such as filters and power dividers have critical specification requirements. These specification requirements include low insertion loss (IL), sharp filter response, also known as high roll-off rate (ROR), compact size, high selectivity, high isolation between output ports, wide stopband response, high suppression factor (SF), simple structure and affordable manufacturing processes. Microwave components which exhibit these attributes play a crucial role in such modern systems.

Microstrip filters such as bandpass filters (BPFs)^4,5^ and lowpass filters (LPFs)^6–15^ have a key role in the design of modern communication systems as they are used for removing unwanted signals and harmonics from radio signals. In^4^, a BPF with compact size and low IL designed using coupled line resonators and spoof surface plasmon resonators for communication applications was presented. Also, recent challenges and techniques for designing BPF were reported in^5^. Moreover, in state-of-the-art wireless communication circuits, RF signal detectors play an essential role in detecting or demodulating any desirable signals from a modulated signal. A compact tunable LPF with sharp ROR and high selectivity for controlling suppression levels and removing unwanted harmonics for use in envelop detector structures was designed and is described in^6^. In addition, in recent years, various techniques and methods such as microstrip stubs^7,8^, defected ground structure (DGS)^3,9,10^ and T-shaped resonators^11,12^ have been utilised for designing LPF structures and microstrip devices.

Indeed, in^3^, a high-sensitivity microstrip patch sensor antenna is proposed which uses a DGS technology envisaged for permittivity characterisation. A LPF with wide stopband and low IL using modified T-shaped hairpin resonators has been presented in^12^. Moreover, a compact LPF with sharp ROR and wide stopband using a stepped impedance resonator is proposed in^13,14^. In addition, in^14^, an ultra-wide stopband LPF with low IL (less than 0.5 dB) using three-stepped impedance stubs and multiple transmission zeros was reported. Although a compact LPF presenting a good response in terms of a sharp ROR, wide stopband and low IL is presented in^15^, the structure is comparatively complex. Combiners, couplers and power dividers are three microstrip passive devices which can be considered essential in modern wireless communication systems for the distribution and processing of RF energy. The Gysel power divider^16,17^ and Wilkinson power divider (WPD)^18–32^ are very common and useful in RF/microwave communication circuits to divide an input signal into two equal phase output signals, or to combine two equal-phase signals into a combined signal with a 180° phase shift. Based on rapid developments in state-of-the-art communications, the demand for multi band frequency components has increased dramatically in recent years^18,19^. However, conventional WPDs have a large footprint, and small stopband bandwidth requiring additional measures such as coupled-line^20,21^, integrating filters and suppression cells^21–32^ to mitigate these issues. A wideband BPF power divider with a suppression cell using microstrip stubs and coupled lines was presented in^21,24^. In recent years, several techniques such as microstrip stubs^22,23,25^, microstrip electromagnetic bandgap elements^26^, bent stub and rectangular resonator^27^, asymmetric spiral DGS^28^, high-low impedance resonators^29^, SMD inductors and resistors^30^ and radial open stubs^31,32^ have been utilised for designing harmonic suppression structures. In modern communication systems, the demand for multiplexer and multi-port components such as power dividers^16–32^ and diplexers^34–38^ has been growing rapidly. In addition, designing LPF, BPF and tunable microstrip devices based on LC circuits and analytical methods in^34–38^ can be very useful. Also, all LPF and BPF parameters such as ROR, SF, normalised circuit size (NCS), figure of merit (FOM), relative stopband (RSB), tuning range (TR), cut-off frequency (*f*_c_) and center frequency (*f*_0_) are reported in^34,35,38^. The design of a WPD that offers harmonic suppression, compact size, wide stopband performance, sharp ROR, high SF, low-cost implementation, with high isolation between output ports, still remains a challenge at the present time.

**Figure 1:**
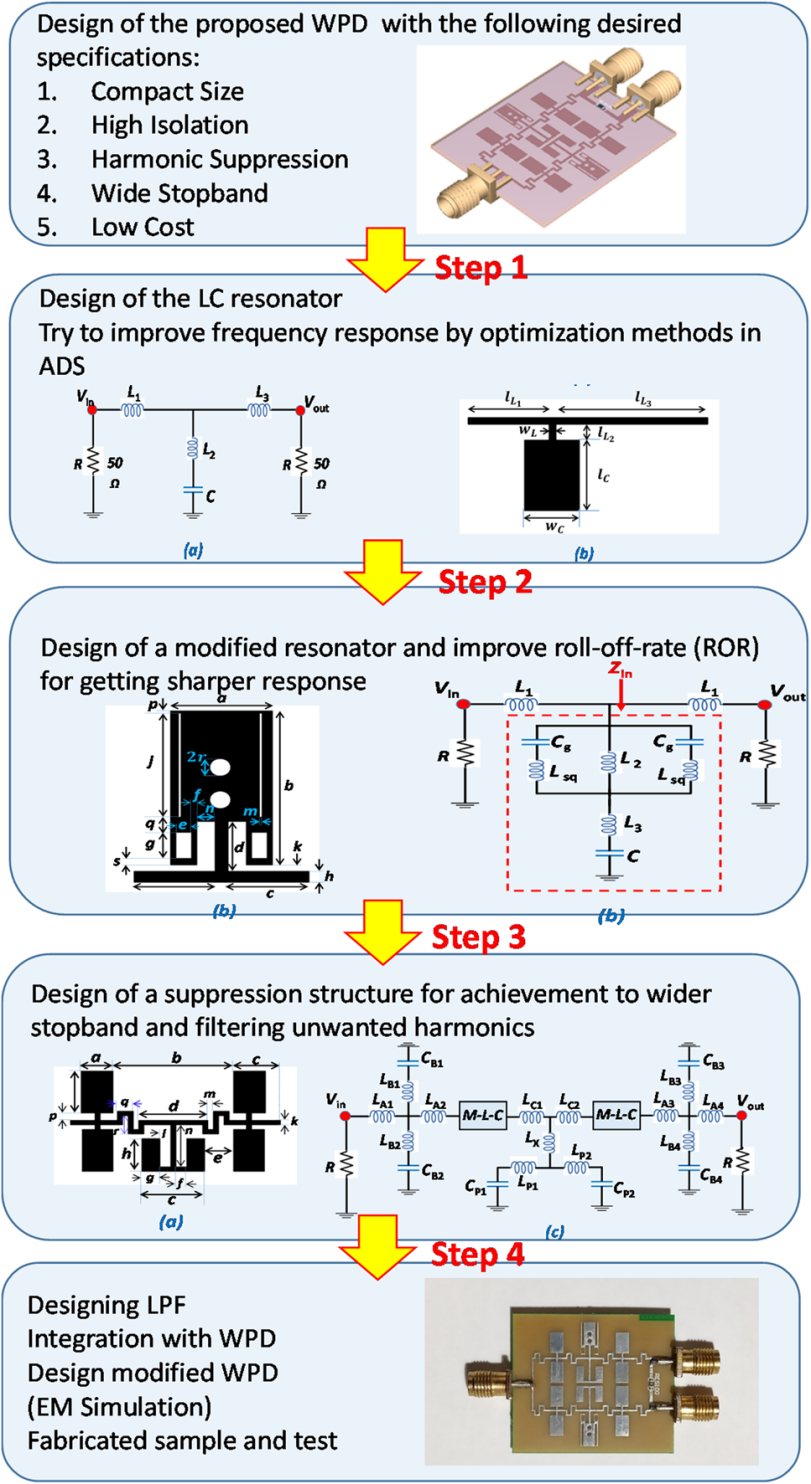
The design procedure of the proposed WPD.

In this paper, a WPD with a modified structure is proposed in “Power divider structure design”. The design steps associated with the proposed modified WPD is shown in Fig. 1. The presented WPD is designed according to desirable characteristic constraints such as size reduction, high isolation, sharp ROR and wide stopband performance. The design uses LPF structures that are integrated into both branches of a conventional power divider. The remainder of the paper is organised as follows: Designing of the third-order LC circuit, basic and modified resonators, harmonic suppression cells and LPF structure are presented in “LPF design methodology”. Conventional and modified WPD and odd and even modes of the proposed WPD are discussed in “Power divider structure design”. Discussion of simulation and measurement results, novelty and advantages of the proposed WPD are explained in “Discussion of results and contributions”. Finally, “Conclusion” presents a conclusion of this work.

## LPF design methodology

**Figure 2:**
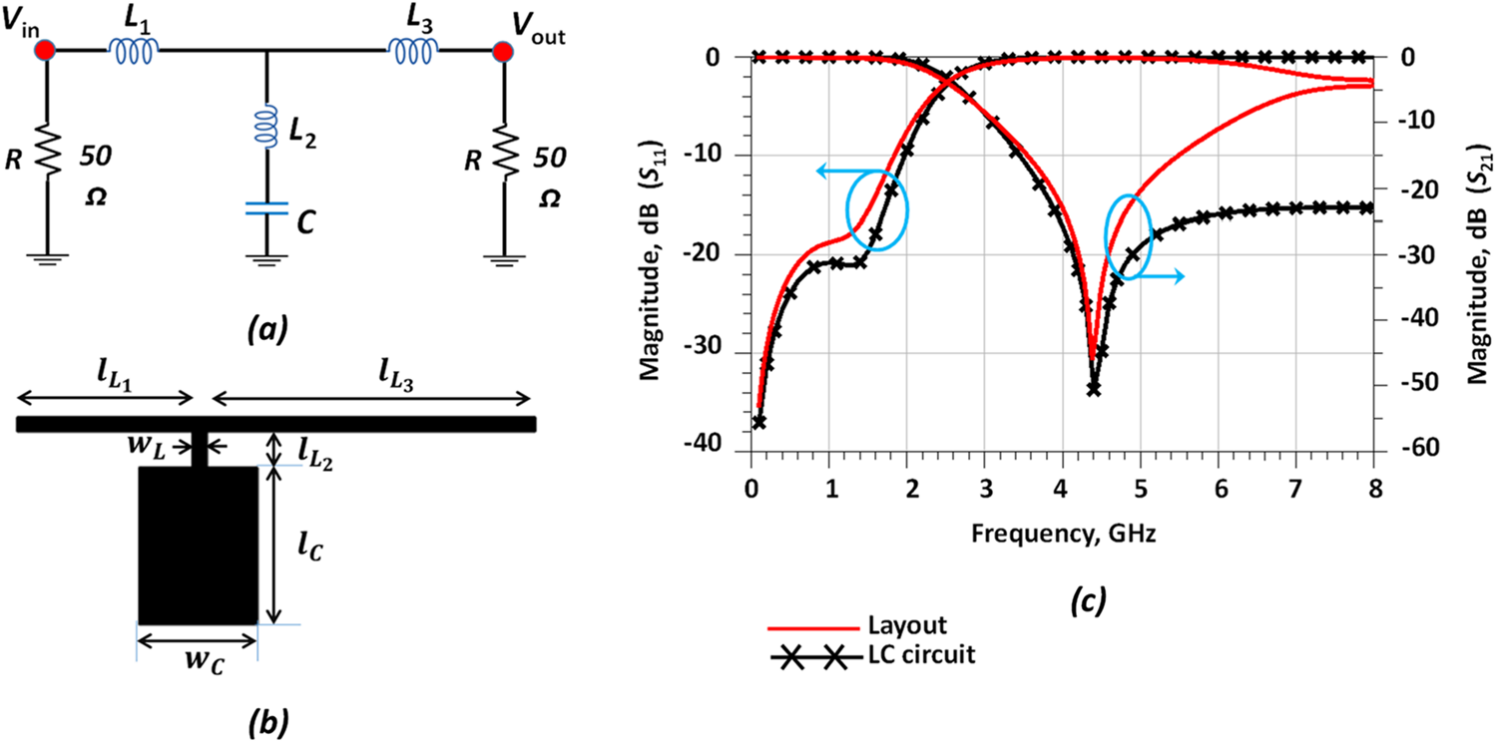
Proposed basic resonator. (**a**) Third-order circuit. (**b**) Layout. (**c**) Simulation results.

**Figure 3:**
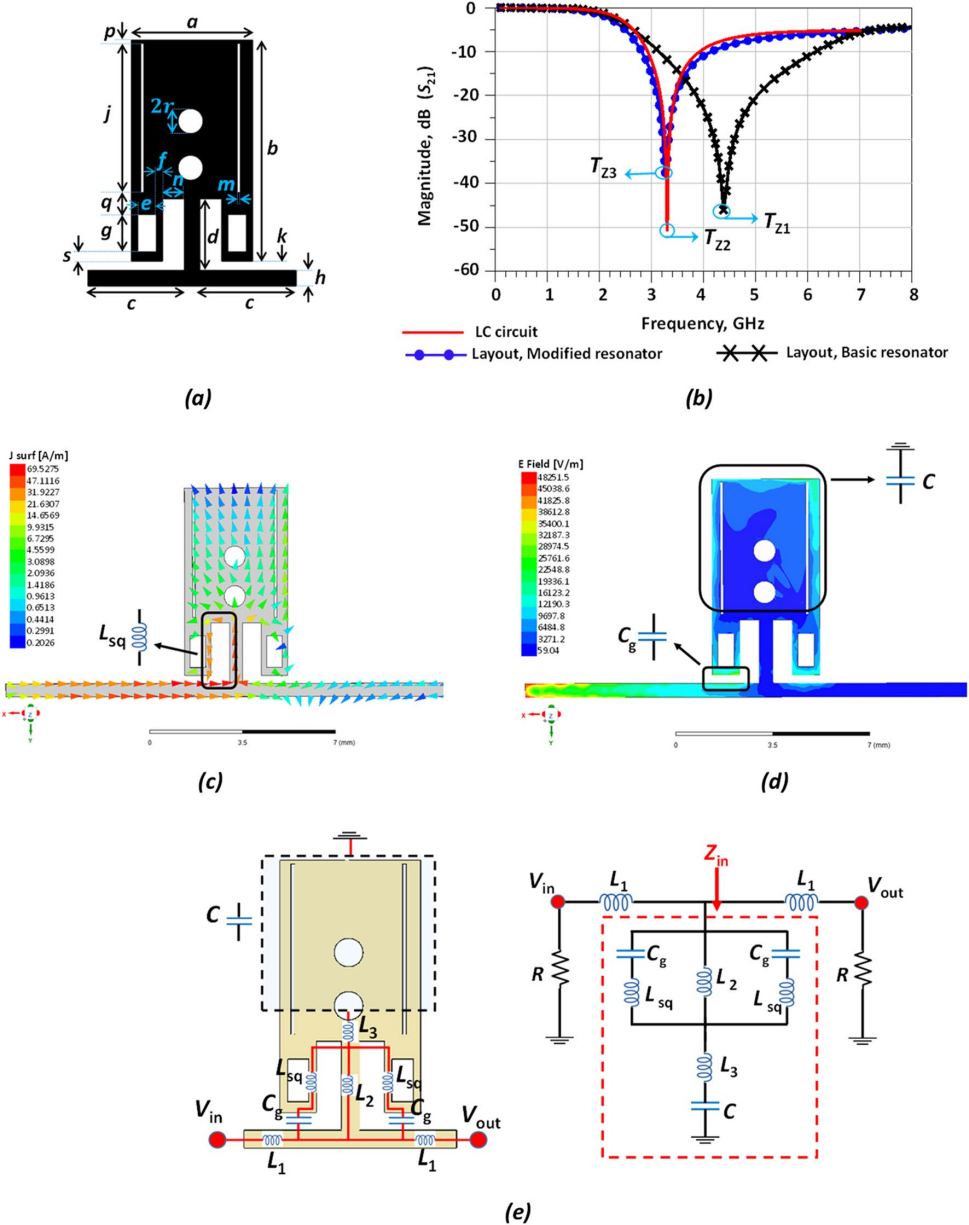
Proposed modified resonator. (**a**) Layout. (**b**) Simulated results. (**c**) Full wave surface current at Tz. (**d**) Electrical field strength at Tz and (**e**). LC equivalent circuit.

**Figure 4:**
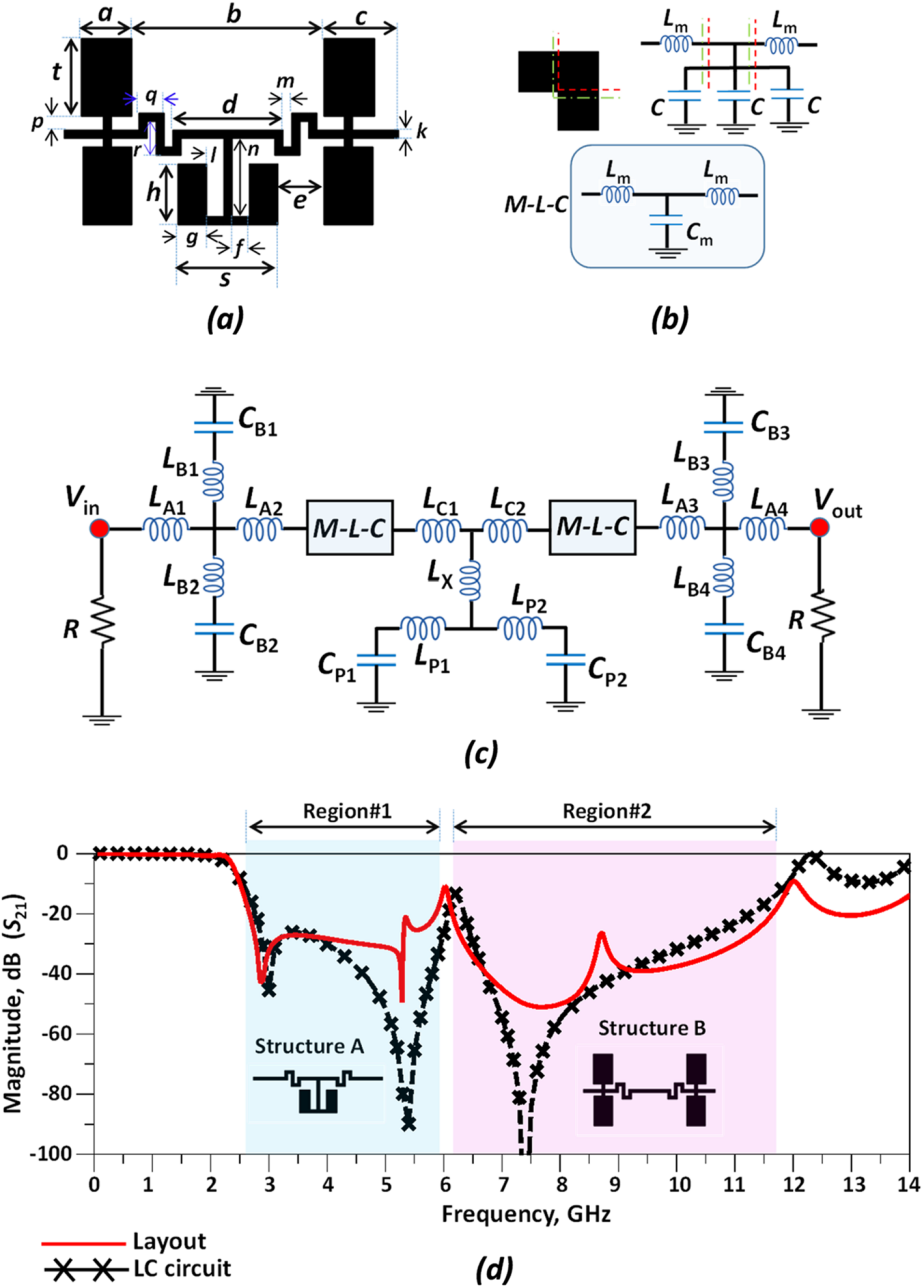
The suppression cell structure. (**a**) Layout. (**b**) Bending line. (**c**) Equivalent LC-circuit and (**d**) simulation results. (The optimized values of the LC-circuit are obtained as: *L*_A1,2,3,4_ = 0.55 nH, *L*_C1,2_ = 1.2 nH, *L*_P1,2_ = 1.2 nH, *L*_B1,3_ = 0.84 nH, *L*_B2,4_ = 1.6 nH, *L*_m1,2,3,4_ = 1.1 nH, *L*_X_ = 0.9 nH, *C*_P1,2_ = 0.95 pF, *C*_m1,2_ = 0.12 pF and *C*_B1,2,3,4_ = 0.55 pF).

**Figure 5:**
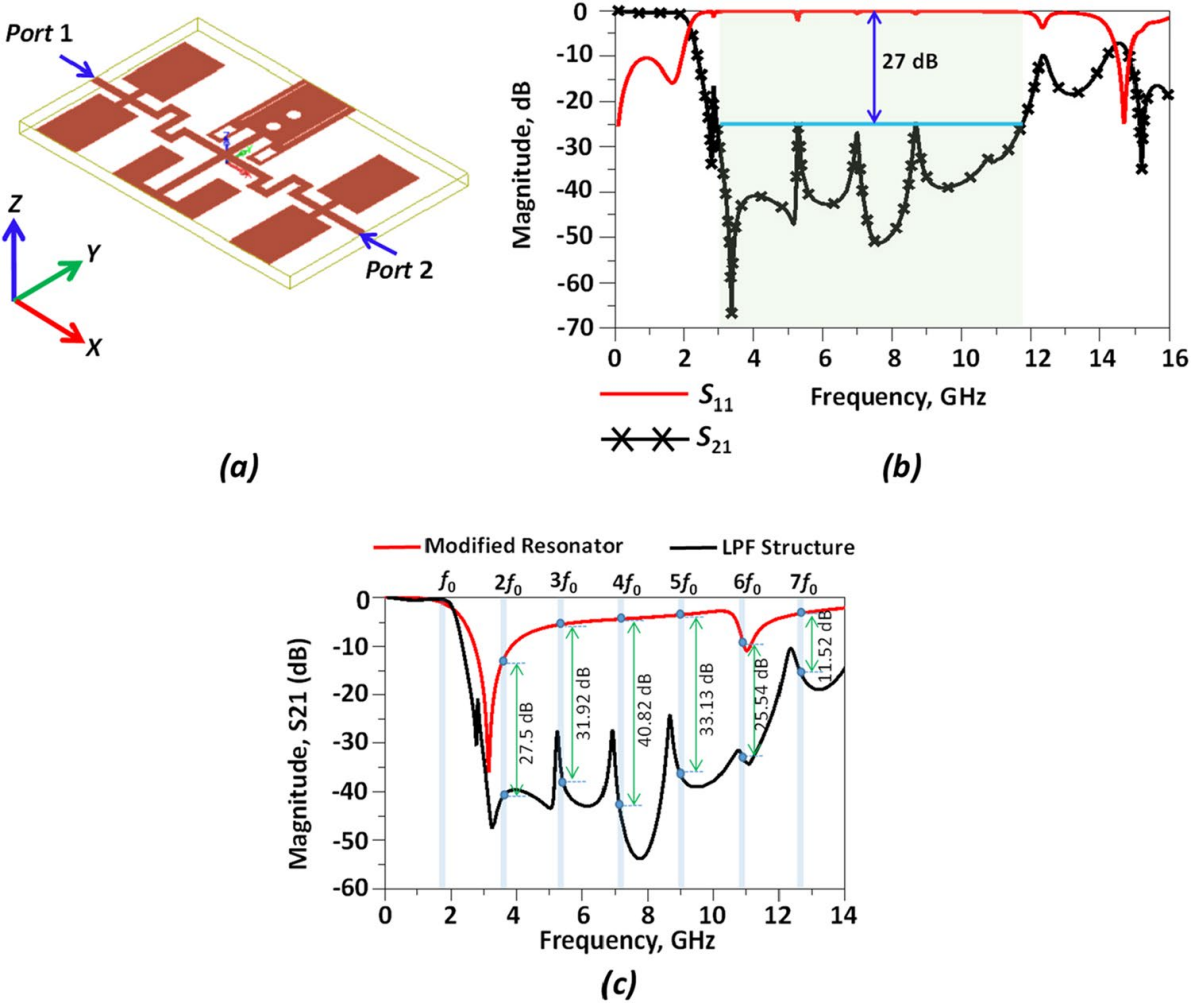
Proposed LPF structure. (**a**) 3D PCB Layout. (**b**) Simulated results. (**c**) Simulated results for the proposed LPF structure and modified resonator. The *f*_0_ (1.8 GHz) is the operating frequency for the proposed WPD.

The LPF design methodology is depicted in Fig. 1. As seen in the Figure, as a first step, a basic LC resonator is designed based on normalization coefficients of the third-order LPF circuit followed by a microstrip transmission line implementation derived from a detailed mathematical analysis (Fig. 2). In the second step, a design methodology for the modified resonator is presented. One of the important performance parameters for a filter is the roll-off-rate (ROR) which depicts the changing attenuation rate from the passband to the stopband. In order to sharpen the LPF response, some novel modifications have been introduced in this work in comparison to a traditional (basic) resonator described extensively in the literature and are depicted in Fig. 3. In fact, the modified resonator has the same fundamental behavior as the traditional basic resonator, with an improved response due to the novel modifications and optimizations that have been included in a simple symmetric structure as shown in Fig. 3. A novel equivalent LC circuit is presented and analyzed for this resonator. According to Fig. 1, in the third design step, a novel harmonic suppression is presented and analyzed. The proposed novel harmonic suppression is shown in Fig. 4 to achieve a wide stopband and suppress harmonics. Then, by adding the proposed novel harmonic suppression to the proposed novel modified resonator an LPF structure is fabricated in Fig. 5. In the final step of Fig. 1, the proposed LPF structure is integrated in both branches of conventional power divider to pass DC frequency and fundamental harmonic and omit other harmonics (2nd to 7th harmonics).

In the first design step, a compact lowpass resonator (LPR) based on the third-order LPF circuit is designed. This LPR is then used as part of a high-performance filter that is integrated into the WPD. The dimensions of the LPR are calculated by normalization coefficients of the third-order LPF circuit with *g*_L1_ = 1.299, *g*_L2_ = 0.0250, *g*_L3_ = 2.142 and *g*_C2_ = 1.344^39^. The values of the capacitors and inductors of the third-order circuit can be obtained using Eqs. (1a) and (1b). Moreover, the physical lengths of the low- and high-impedance lines are calculated using Eqs. (1c) and (1d)^39^.

1a $${L}_{i}= \frac{1}{{2\pi f}_{c}} {Z}_{0} {g}_{Li}$$

1b $${C}_{i}= \frac{1}{{2\pi f}_{c}} \frac{1}{{Z}_{0}} {g}_{Ci}$$

1c $${l}_{Li}= \frac{{\uplambda }_{gLi}}{2\pi } {Sin}^{-1}\left(\frac{{2\pi f}_{C}{L}_{i}}{{Z}_{0L}}\right)$$

1d $${l}_{Ci}= \frac{{\uplambda }_{gCi}}{2\pi } {Sin}^{-1}\left({2\pi f}_{C}{C}_{i}{Z}_{0C}\right)$$

In the above equations, *Z*_0_ is the characteristic impedance of the transmission line (50 Ω). The circuit configuration and PCB layout of the LPR and third-order circuit are depicted in Fig. 2. The values of the LC third-order circuit are *L*_1_ = 3.864 nH, *L*_3_ = 2.65 nH, *C* = 1.71 pF, and *L*_2_ = 0.754 nH. The parameters *λ*_gL_ and *λ*_gC_ are the corresponding guided wavelengths, and *Z*_Li_ and *Z*_Ci_ are the impedances of transmission lines with high and low impedance, respectively. Dimensions of the LPR are specified as *W*_L_ = 0.5, *l*_L1_ = 6.067, *l*_L3_ = 11.351, *l*_L2_ = 1.035, *W*_C_ = 4 and *l*_C_ = 4.652 (all dimensions in mm). In order to achieve an improved filtering response (sharper ROR), the LPR needs to be optimised. This can be accomplished using built-in optimisation methods in ADS, or alternatively, implementing custom-made optimisation algorithms. Here, the ADS optimisation option was employed, and LPR dimensions were updated as follows: *W*_L_ = 0.5, *l*_L1_ = 5.8, *l*_L3_ = 11.1 and *l*_L2_ = 1.2; for capacitors: *W*_C_ = 4 and *l*_C_ = 5.3 have been obtained (all dimensions in mm).

According to Fig. 1, the first objective is to design a LPF structure (by using a modified resonator and harmonic suppression cells), and the primary design constraint is the integration of a LPF into the WPD branches to eliminate harmonics. According to Fig. 2c, a good agreement is observed between the simulation results of the layout and the LC third-order circuit. The ABCD parameters of the proposed resonator (third-order LPF circuit) are obtained as:

1e $$A= \frac{1- {\omega }^{2 }{C}_{ }({L}_{1}+ {L}_{2} )}{1- {\omega }^{2 }{C}_{ }{L}_{2}}$$

1f $$B =\frac{\left[\begin{array}{c}{j\omega }^{3 }{C}_{ }{L}_{2}{L}_{3 }-j\omega \left({L}_{3}+{L}_{1 }\right)+\\ j{\omega }^{3 }C {L}_{1}\left({L}_{2}+{L}_{3}\right)\end{array}\right] }{1- {\omega }^{2 }{C}_{ }{L}_{2} }$$

1g $$C= \frac{j \omega C}{1- {\omega }^{2 }C {L}_{2}}$$

1h $$D= \frac{1- {\omega }^{2 }C({L}_{3}+ {L}_{2} )}{1- {\omega }^{2 }C {L}_{2}}$$

The relationship between Insertion Loss (IL) (*S*_21_ parameter) the ABCD parameters referred to in Eq. (1e) to (1h) can be expressed as:

2a $${S}_{21}= \frac{2{Z}_{0} (1-{\omega }^{2} C {L}_{2})}{{Z}_{0} {K}_{1 }+{K}_{2 }+{K}_{3}}$$

2b $${K}_{1}=\left[2-{\omega }^{2}C\left({L}_{1}+{L}_{3}+{2L}_{2}\right)\right]$$

2c $${K}_{2}=j{\omega }^{3}C\left({L}_{2}{L}_{3}+{L}_{1}{L}_{2}+{L}_{1}{L}_{3}\right)$$

2d $${K}_{3}=j\omega \left(C-{L}_{3}-{L}_{1}\right)$$

According to Eq. (2a), the location of the transmission zero (*T*z) can be controlled by parameters *C* and *L*_*2*_.

3 $${T}_{z}\to 2{Z}_{0} \left(1-{\omega }^{2} C {L}_{2}\right)=0$$

$${T}_{Z}=\frac{1}{2\pi \sqrt{{L}_{2}C}}=\frac{1}{2\pi \sqrt{0.754*{10}^{-9}*1.71*{10}^{-12}}}$$

4 $${T}_{Z}=4.486 \mathrm{GHz}$$

The transition function for the third-order circuit is defined by Eq. (5).

5 $$\frac{{v}_{out}}{{v}_{in}}=\frac{ R\left(1+C{L}_{2}{S}^{2}\right) }{ \left[\begin{array}{c}R+{L}_{1}S+{L}_{3}S+C {L}_{1}{R S}^{2}\\ +C {L}_{2}{R S}^{2}+C {L}_{1}{{L}_{2} S}^{3}+\\ C {L}_{1}{{L}_{3} S}^{3}+C {L}_{3}{{L}_{2} S}^{3}\end{array}\right] }$$

According to Eq. (5), the location of the transmission zero or *T*z can be controlled by parameters *C* and *L*_2_.

6 $${T}_{z}\to R \left(1+{S}^{2} C {L}_{2}\right)=0$$

$${T}_{Z}=\frac{1}{2\pi \sqrt{{L}_{2}C}}=\frac{1}{2\pi \sqrt{0.754\times {10}^{-9}\times 1.71\times {10}^{-12}}}$$

7 $${T}_{Z}=4.486\mathrm{ GHz}$$

According to the above equations, there is a good agreement between the *T*z results for the third-order circuit. The comparison between the calculated and simulated results for *T*z is illustrated in Table 1.

**Table 1:** Comparison between calculated and simulated results for *T*z for the basic resonator.

*T*z (GHz)	Equation (4)	Equation (7)	Simulation LC-circuit and layout
Tz(GHz)	4.486	4.486	4.462

The sharp response (response with fast ROR) is one of the important parameters of the LPF structures that can be calculated by the following equation.

8 $$ROR= \frac{{\alpha }_{max} - {\alpha }_{min}}{{f}_{s }- {f}_{c}}\left(\frac{\mathrm{dB}}{\mathrm{GHz}}\right)$$

In (8), *α*_max_ is usually − 40 dB (or − 20 dB) and *α*_min_ is − 3 dB; *f*_s_ is the frequency of − 40 dB point, and *f*_c_ is the cut-off frequency. In order to sharpen the LPR response, some novel modifications are introduced to the basic resonator and are depicted in Fig. 3. The novel equivalent LC circuit of the modified LPR is also depicted to provide a better insight into the filtering mechanism.

According to the Fig. 3b, the ROR parameter for the basic resonator and modified resonator are obtained as 18.28 dB/GHz and 61.15 dB/GHz, respectively. The location of *T*z for the basic and modified resonators are in the vicinity of 4.48 GHz (with 44.86 dB) and 3.27 GHz (with 38.48 dB), respectively. The dimensions of the modified resonator have been optimized in ADS. The optimized length and width values for the modified resonator are: *a* = 3.9, *b* = 7.1, *c* = 3.1, *d* = 2.3, *e* = 0.6, *f* = 0.2, *g* = 1.2, *h* = 0.5, *j* = 4.8, *k* = 0.3, *m* = 0.1, *n* = 0.7, *p* = 0.1, *q* = 0.7, *r* = 0.4 and *s* = 0.3 (all in mm). The input impedance (*Z*_in_) for the main branch of the equivalent LC circuit (depicted in Fig. 3e) is calculated in Eq. (9).

9 $${Z}_{in}=\frac{ \alpha {S}^{4}+\beta {S}^{2}+1}{ SC\left[{S}^{2}\left({C}_{g}{L}_{sq}+{L}_{2}{C}_{g}\right)+1\right] }$$

10 $$\alpha ={L}_{sq} {C}_{g}{L}_{2}C+{L}_{3} {C}_{g}{L}_{sq}C+2{L}_{3}{C}_{g}{L}_{2}C$$

11 $$\beta ={L}_{2}C+{L}_{3}C+{L}_{sq}{C}_{g}+2 {L}_{2}{C}_{g}$$

According to Eq. (12), the location of *T*_Z_ can be obtained by the following equations:

12 $${T}_{Z}=\pm \sqrt{\frac{1}{4{\pi }^{2}}} \times \sqrt{\frac{\beta \pm \sqrt{k} }{2C{C}_{g}{(2L}_{2}{L}_{3}+{L}_{2}{L}_{sq}+{L}_{3}{L}_{sq}) }}$$

13 $$K=\left[{C}^{2}{{L}_{2}}^{2}+2{C}^{2}{L}_{2}{L}_{3}+{C}^{2}{{L}_{3}}^{2}+4C{C}_{g}{{L}_{2}}^{2}-4C{C}_{g}{L}_{2}{L}_{3}-2C{C}_{g}{L}_{2}{L}_{sq}-2C{C}_{g}{L}_{3}{L}_{sq}+4{{C}_{g}}^{2}{{L}_{2}}^{2}+4{{C}_{g}}^{2}{L}_{2}{L}_{sq}+{{C}_{g}}^{2}{{L}_{sq}}^{2}\right]$$

The inductance and capacitance values for the equivalent LC circuit for the modified LPR were optimized to *L*_1_ = 1.2 nH, *L*_2_ = 0.6 nH, *L*_3_ = 1.5 nH, *C*_g_ = 0.2 pF, *L*_sq_ = 0.4 nH and *C* = 1.2 pF. According to Table 2, there is a small difference in the results between the *T*z and modified LPR. The comparison between the calculated and simulated results for *T*z is depicted in Table 2.

**Table 2:** Comparison between calculated and simulated results for the *T*z of the modified resonator.

Tz	Equation (12)	Layout	Simulation LC-circuit
	3.125 GHz	3.271 GHz	3.127 GHz

A more accurate equivalent circuit of the proposed resonator is extracted based on the EM simulations. The surface current and electrical field distribution are shown in Fig. 3c,d to clarify the proposed LC circuit in Fig. 3e. According to the above explanation, LC circuit and layout for the basic resonator are conventional, but they have been used for creating a novel modified resonator. Moreover, the proposed modified resonator and presented LC circuit based on surface current and electrical field in a full wave simulation are more accurate, symmetric and novel.

To achieve a wide stopband and improve the frequency response in the rejection band, a suppression structure has been added to the modified LPR. The suppression structure is shown in Fig. 4. The proposed suppression cell consists of two structures (structures A and B) regarding to the Fig. 4d to suppress *S*_21_ in the rejection band in Region#1 and region#2, respectively and creating a wide stopband. The suppressor cell causes pulldown of the IL parameter in the stopband in the range of 2.65–5.88 GHz (Region#1 in Fig. 4) and 6.16–11.68 GHz (Region#2 in Fig. 4) with more than 20 dB attenuation level. The length and width values of the suppressor cell are: *a* = 3, *b* = 11.3, *c* = 4.4, *d* = 6.5, *e* = 2.7, *f* = 1, *g* = 1.7, *h* = 3.6, *k* = 0.5, *l* = 1, *m* = 0.5, *n* = 4.6, *p* = 0.8, *q* = 1.5, *r* = 2, *s* = 5.9 and *t* = 4.6 (all in mm). The layout, equivalent LC circuit and simulation results for the proposed suppression cell structure are illustrated in Fig. 4. According to the Fig. 4d, a good agreement is observed between the layout and equivalent LC circuit results. In order to create a wider stopband, more suppression structures are required which results in a sophisticated LC circuit with a complex structure, so there is a tradeoff between the number of suppression structures and creating a wider stopband. The proposed LPF structure is designed and analyzed in the remainder of this section.

The introduction of the suppressor cell to the modified LPR yields a LPF with a sharp response and large stopband as depicted in Fig. 5. Table 3 illustrates the comparison of the proposed LPF with some of the recent filters in the literature from the same class. The − 3 dB cut-off frequency for the proposed LPF is 2.16 GHz. The frequency response (ROR parameter) is sharp and equal to 53 dB/GHz. The transition band is narrow and covers a frequency band from 2.16 to 2.76. The IL and RL in the passband and stopband are better than 0.4, 10.6, 20 and 0.3 dB, respectively. The suppression level across 90% of the stopband is better than 27 dB leading to SF parameter being 2.7. The stop bandwidth is also desirably large and covers a 9.1 GHz range (2.88–11.98 GHz), with an RSB of 1.23 having been obtained.

**Table 3:** Comparison between simulated results and other studies for LPF.

References	*f*_c_ (GHz)	ROR (dB/GHz)	RSB	SF	Size (*λ*g^2^)	IL (dB)
^7^	1.9	37	1.45	1.8	–	0.3
^9^	1.9	85	1.43	2.5	0.31 × 0.21	0.5
^10^	1	78	–	2	0.16 × 0.1	0.3
^11^	2.68	42.5	1.51	2	–	0.12
^14^	0.9	80			0.17 × 0.10	0.5
^15^	2	123	1.65	2	–	0.3
This work	2.16	53	1.23	2.7	0.24 × 0.16	0.4

After designing the modified resonator, a suppression cell structure is presented in Fig. 4 which is envisaged to create a wider stopband for the proposed LPF structure. In fact, the final LPF structure (in Fig. 5a) consists of the modified resonator and the suppression cell structure. According to the Fig. 5c, there is a significant reduction in the harmonic levels (for 2nd to 7th harmonics). According to this figure, all the unwanted harmonics (2nd to 7th) have been located in the stopband region of the proposed LPF. The operation for the proposed LPF structure developed to suppress harmonics has been illustrated in Fig. 5c. The proposed LPF structure will be integrated in both branches of a conventional WPD to create a wide stopband and suppress harmonics in the next section.

## Power divider structure design

**Figure 6:**
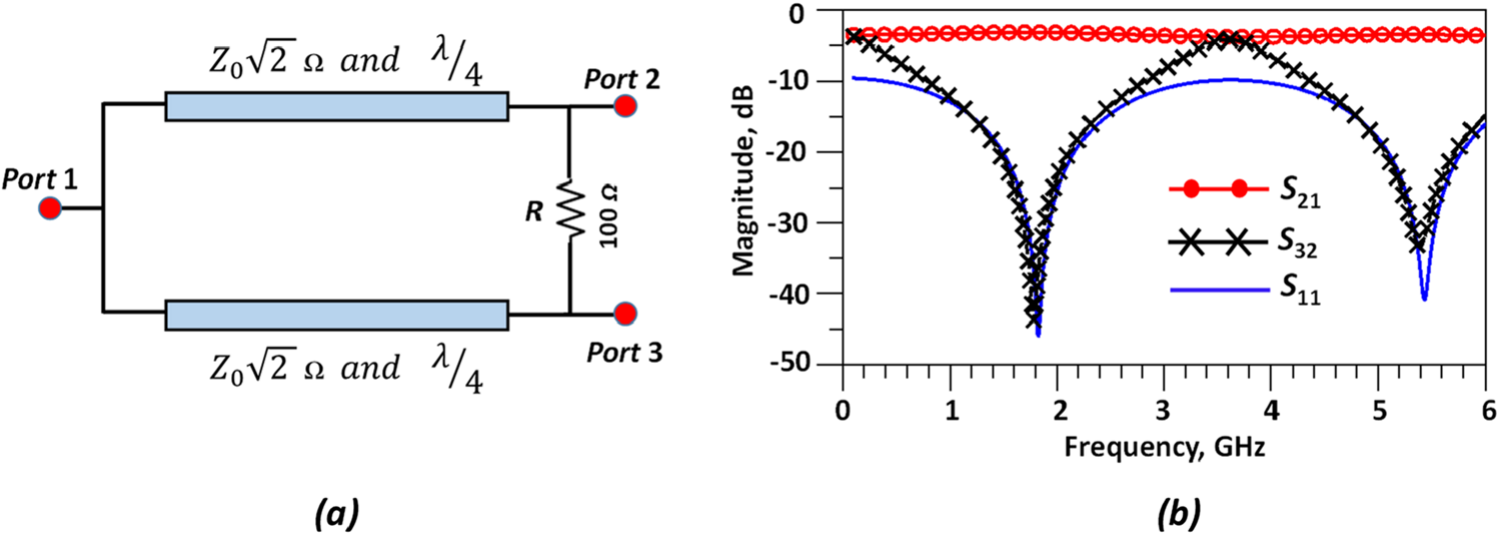
Conventional WPD. (**a**) Schematic and (**b**) simulation results.

**Figure 7:**
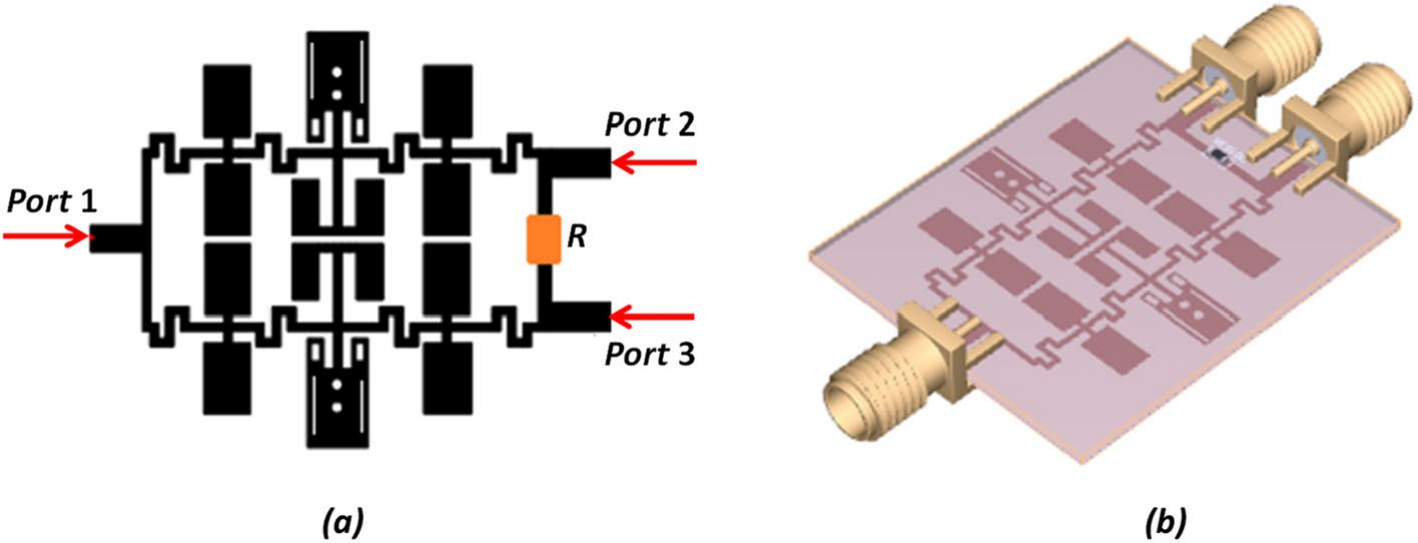
Modified WPD. (**a**) PCB Layout and (**b**) 3D-Schematic.

A conventional microstrip WPD is designed by implementing two quarter wave-length microstrip lines (*λ*/4 transmission lines) with a characteristic impedance of 70.7 Ω (${{\varvec{Z}}}_{0}\sqrt{2}\boldsymbol{ }\boldsymbol{\Omega }$ and a 100 Ω terminating resistor. A schematic for the block diagram and simulation results of the proposed conventional WPD are shown in Fig. 6. According to the simulation results, the input return loss (*S*_11_), insertion loss (*S*_21_) and isolation (*S*_32_) at 1.8 GHz are better than 46.85 dB, 3.17 dB and 44.72 dB, respectively. The proposed LPF structure is integrated into both branches of conventional power divider as a means for improving harmonic suppression and achieving a sharper response with a wider stopband. The proposed modified WPD is depicted in Fig. 7.

**Figure 8:**
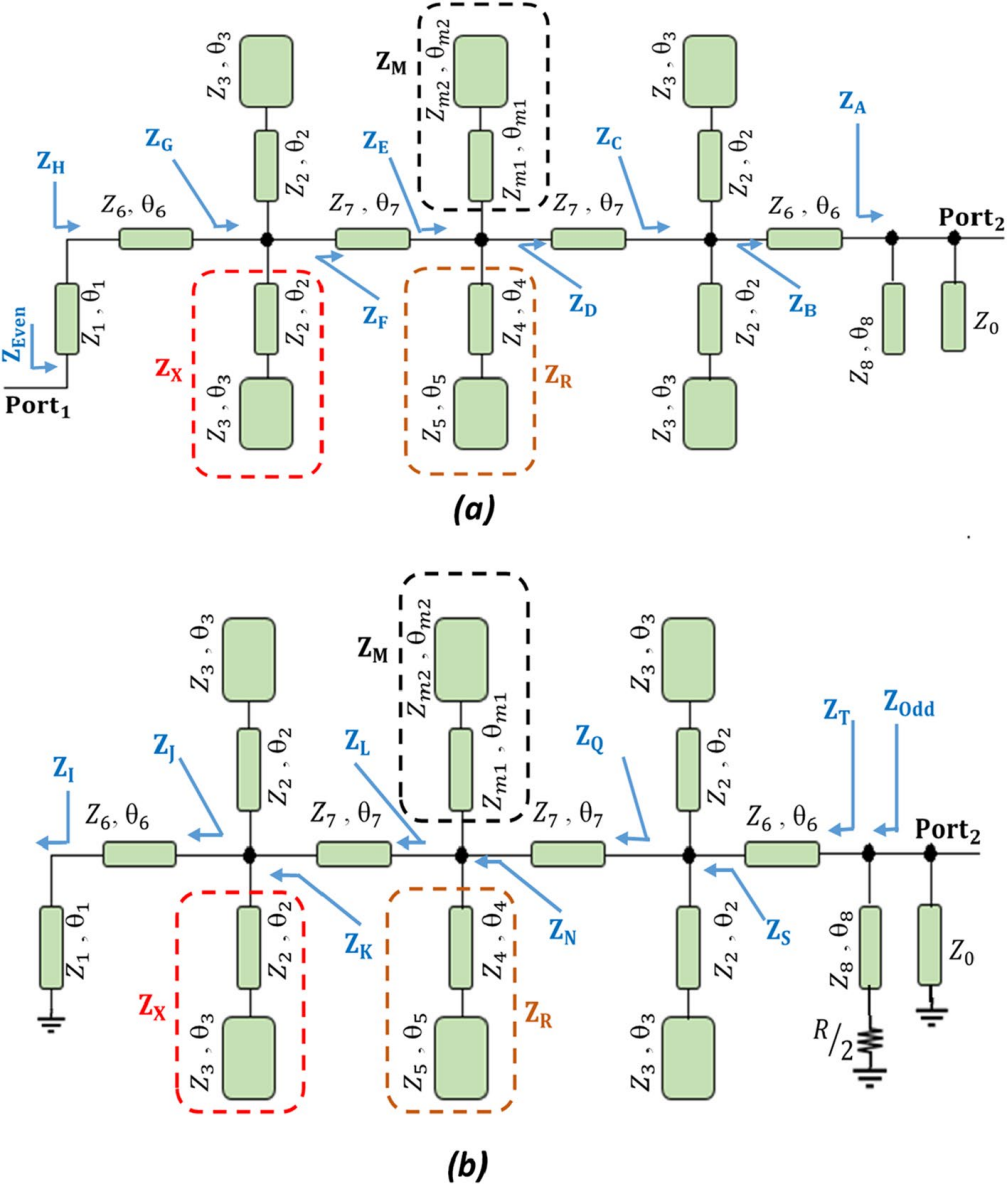
Equivalent circuit of the modified WPD. (**a**) Even mode and (**b**) odd mode.

The even and odd mode circuits which form the critical building blocks for the modified WPD are illustrated in Fig. 8. The expressions for calculating the even and odd mode parameters are described in detail in Appendix (Additional/Supplementary Information)

## Discussion of results and contributions

**Figure 9:**
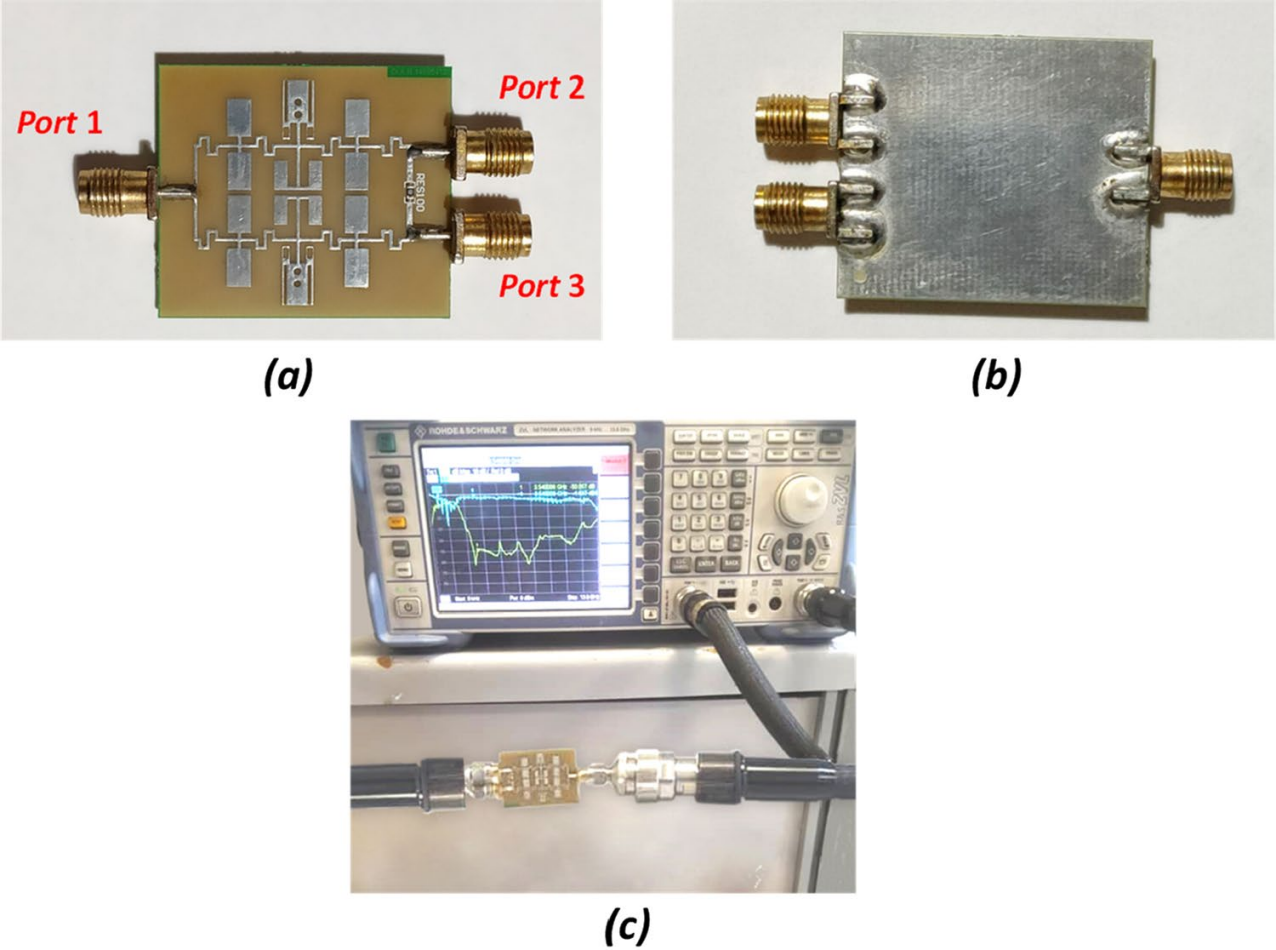
Fabricated sample of modified WPD. (**a**) Top, (**b**) bottom, (**c**) test setup.

The proposed power divider has been implemented on a low-cost FR4 substrate with a dielectric constant of 4.4, a thickness of 0.8 mm (~ 31 mil) and a loss tangent 0.022. The fabricated prototype WPD and measured results are depicted in Fig. 9.

**Figure 10:**
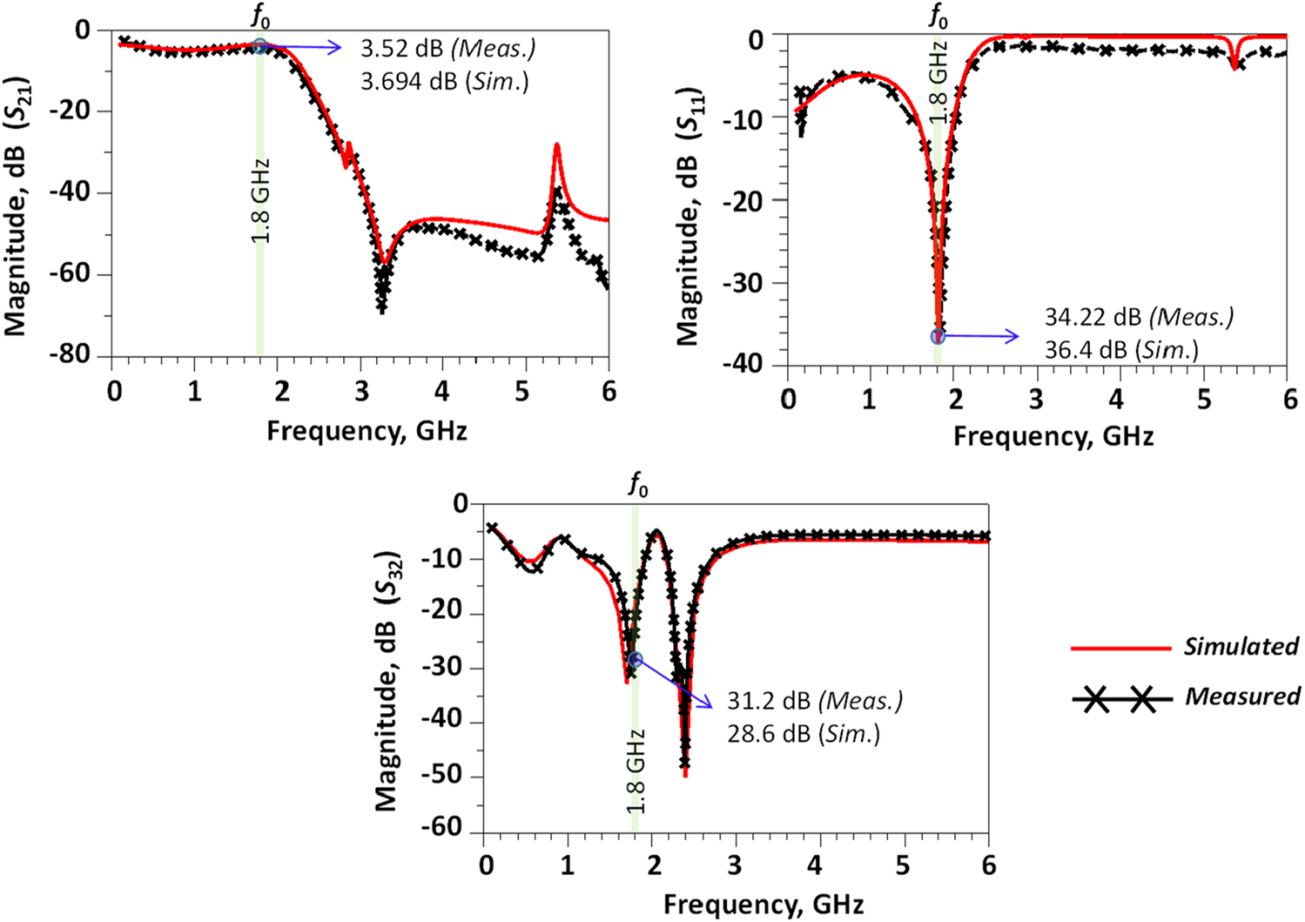
Simulated and measured results (0.1–6 GHz) for the proposed modified WPD.

The simulated and measured results for the modified WPD are shown in Fig. 10 with the frequency varying between 0.1 and 6 GHz. A R&S ZVL13 Vector Network Analyzer was used to measure the *S*-parameters response with a frequency range of 0.1–13.6 GHz during measurements. According to the results, there is a good agreement between the simulated and measured results.

**Figure 11:**
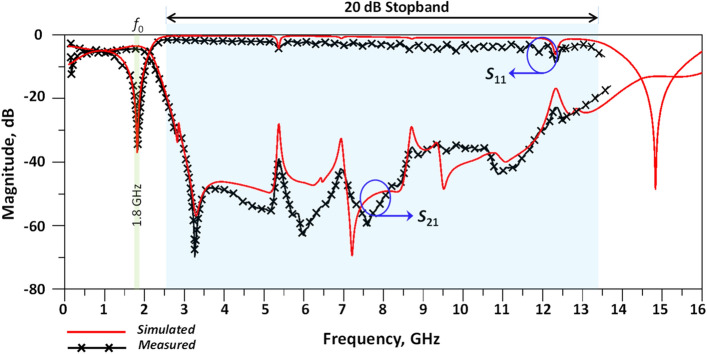
Simulated (0.1–16 GHz) and measured results (0.1–13.6 GHz) for the proposed modified WPD.

The isolation between output ports (|*S*_32_|), input return loss (|*S*_11_|) and insertion loss (|*S*_21_|) are better than 31.2 dB, 34.2 dB and 3.52 dB, respectively, at 1.8 GHz. The simulated results over the frequency range of 0.1–16 GHz and the measured results in the range of 0.1–13.6 GHz are presented in Fig. 11.

**Figure 12:**
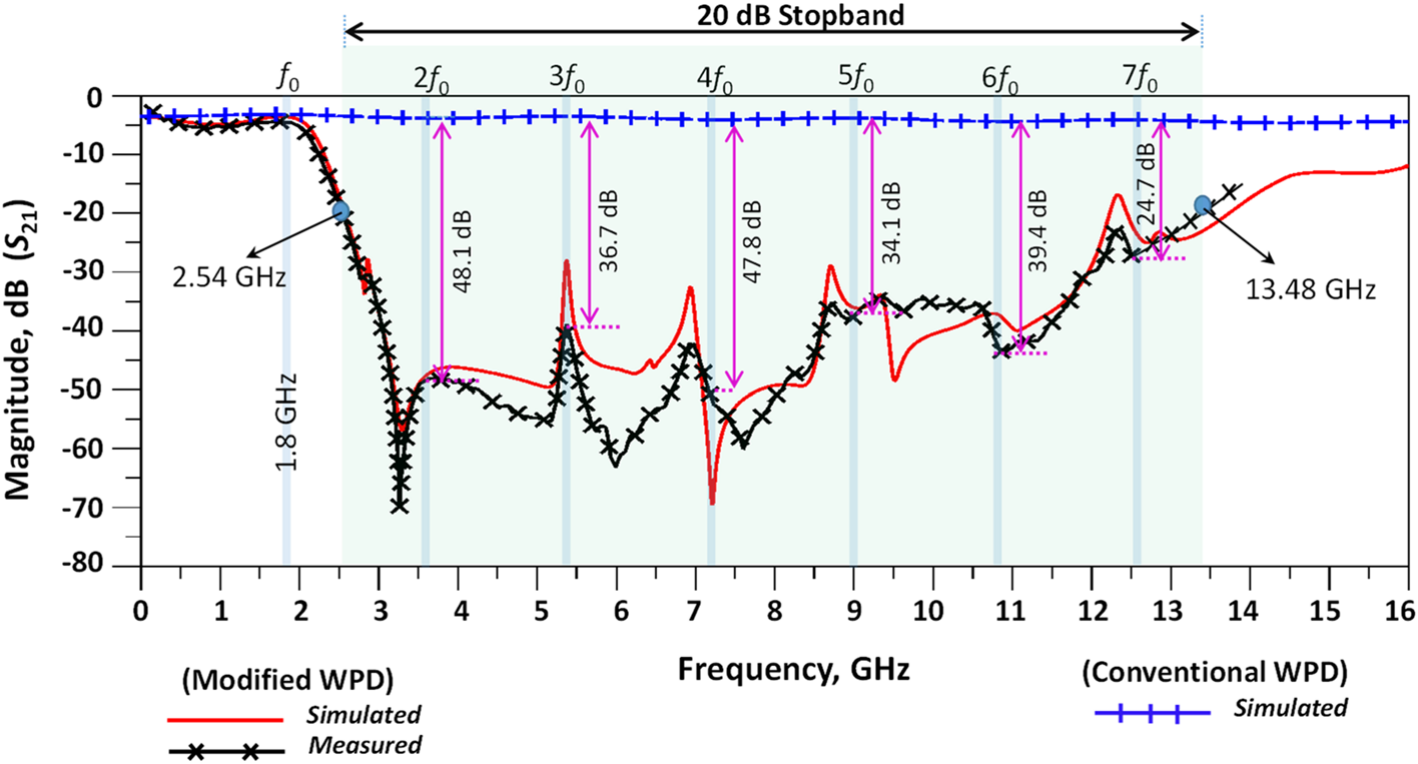
Harmonic suppression, simulation (0.1–16 GHz) and measurement (0.1–13.6 GHz) results of proposed conventional and modified WPDs.

As observed, there is good agreement between the simulated and measured results. Figure 12 shows the harmonic suppression level and stopband bandwidth of the proposed modified and conventional WPDs. According to the results, the proposed WPD has a wide stopband from 2.53 to 12.21 GHz and 2.54 to 13.48 GHz with 20 dB attenuation levels for both the simulated and measured results, respectively. According to Fig. 12, the proposed modified WPD can provide additional attenuation of unwanted signals and harmonics in the stopband region. The second to seventh harmonics at frequencies 2*f*_0_ to 7*f*_0_ (*f*_0_ = 1.8 GHz) are shown to be suppressed with better than 51.2, 40.3, 51.36, 37.5, 42.9 and 28.2 (all in dB) attenuation levels. By comparison with the literature for modified and conventional WPDs, desirable suppression levels in the rejection band are demonstrated for the proposed WPD structure and unwanted signals and harmonics have been greatly attenuated in this region.

**Figure 13:**
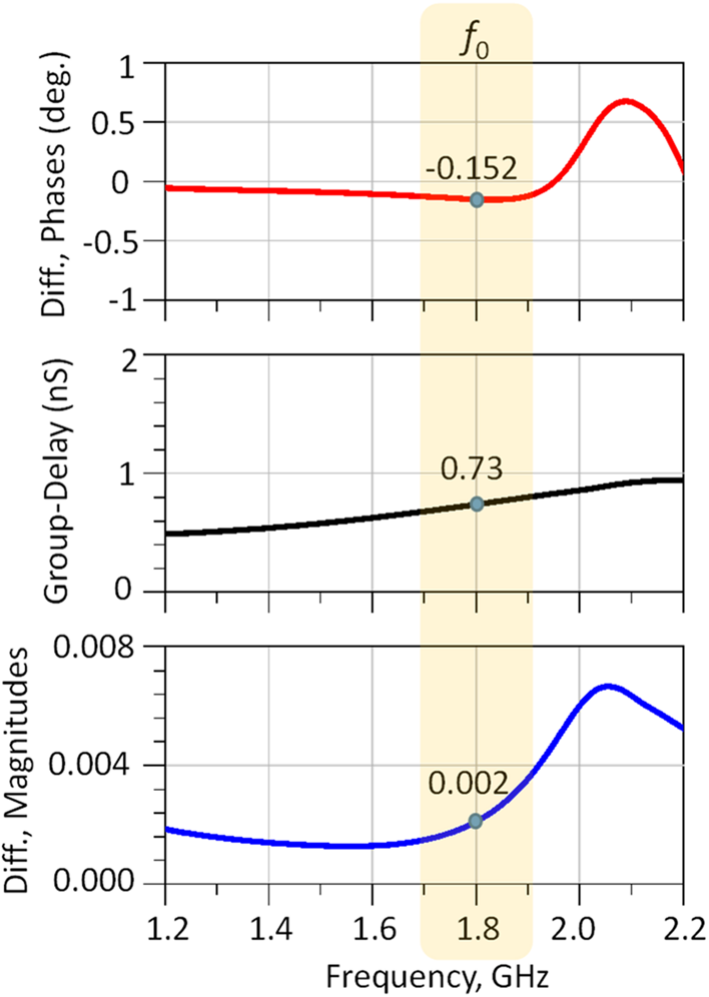
Group delay and magnitude and phase imbalance for the proposed WPD.

Figure 13 plots the simulated values for magnitude and phase imbalance as well as group delay. It can be seen from the results that the modified WPD exhibits a magnitude imbalance of 0.002, a phase imbalance of − 0.152 degree and a group delay of 0.73 ns. These results compare very favorably with the literature and demonstrate the symmetrical nature of the proposed structure.

Table 4 depicts a comparison between the obtained results and other works described in the literature. As can be seen in Table 4, the proposed modified WPD performs well in comparison to other reported works. The output isolation is seen to perform well and shows improved measurements when compared to the isolation results reported in^18–26,28–32,40–42^. The input and output return loss have been obtained and also show an improvement over the results described in such works as^18–43^. In terms of the stopband, the WPD also has a large stopband, extending from 2.54 to 13.48 GHz. This is greater than the stopbands reported in^18–23,26–43^. The presented WPD omits the second to seventh unwanted harmonics with higher suppression levels in comparison with other works^18–21,23,24,26–43^. As can be seen from the comparison table (Table 4), the proposed modified WPD shows superior performance compared to the other works where various WPD technologies have been reported^44–46^. In relation to the novelty of the proposed structure, an LPF with LC equivalent circuits was inserted in both branches of the WPD to realize a novel power divider (PD). The major size of the proposed WPD belongs to the LPF and its dimension is inversely proportional to the frequency of operation and order of the filter. Indeed, a WDP with higher ROR requires higher order filter which consequently increases the size of the LPF. Therefore, there is a trade-off between the size of the structure and the performance of the WPD, particularly with respect to ROR, SF and wide stopband results.

**Table 4:** Comparison of the proposed modified Wilkinson power divider with related works in the literature.

References	*f*_0_ (GHz)	Isolation, *S*_32_ (dB)	*S*_11_ (dB)	*S*_21_ (dB)	*S*_22_ (dB)	Harmonics suppression	Size (*λ*g^2^)	Stopband (GHz)
^18^	1.8	15.2	16.5	4	14.3	–	0.036 × 0.02	–
^21^	2.05	10.2	10.1	3	~ 15	2nd	–	2.98 to 4.93
^22^	0.7	> 28	> 20	3.2	> 25	2nd–15th	0.11 × 0.044	2.9 to 10.5
^23^	1	20	20	3.25	20	2nd–4th	–	1.94 to 4.26
^24^	2.45	11.8	> 25	3.4	–	2nd–7th	0.19 × 0.25	4.8 to 19.92
^25^	1.8	20.1	21.2	3.1	–	2nd-11th	0.31 × 0.16	4.4 to 19.9
^26^	1.8	20	20	3.6	20	2nd-3rd	–	–
^27^	1.8	34.6	20.1	3.1	20	2nd–6th	0.14 × 0.16	3.41 to 10.9
^28^	1.5	–	40	3.2	–	2nd-3rd	–	–
^29^	2.65	22	27	3.4	–	3rd–5th	–	–
^30^	1	20	18	0.52	22	2nd–4th	–	1.8 to 4
^32^	1.5	23.8	19.97	3.22	–	2nd–5th	–	~ 3.3 to 7.9
^43^	1.8	35	34	3.2	–	2nd–7th	0.28 × 0.14	~ 4 to 13.2
^40^	1.74	23	34	3.3	23	2nd–5th	0.11 × 0.13	~ 3.1 to 9.1
^41^	4.11	17	22	3.8	–	–	0.29 × 0.45	–
^42^	1	13.5	30	> 3.4	–	2nd–5th	–	–
^44^	0.9	23	> 20	4.75	> 20	–	0.10 × 0.2	–
This work	1.8	31.2	34.2	3.52	26.2	2nd–7th	0.42 × 0.33	2.54 to 13.48

There are several positive aspects regarding novelty, contribution and design methodology that have been described in detail throughout this paper. In this paper, an effort to establish a novel modified WPD with good performance characteristics using a new, simple and low cost structure for operation at 1.8 GHz in GSM and LTE communication applications has been accomplished and described. To summarise the contributions and strengths of this research work, the benefits of the proposed modified WPD are summarized as follows.

- LPF structure: in this work, a third-order LPF has been designed based on the techniques and equations described in^39^. In the proposed work, a sharper frequency response is achieved using a novel modified resonator with the LC equivalent circuit for all structures and *T*z calculation described in detail.
- Modified WPD structure: although conventional WPDs can provide acceptable results around the center frequency, the out-of-band response is quite poor. In contrast, the WPD presented in this work also demonstrates good performance for the out-of-band performance: in other words, the WPD exhibits a high isolation level, high output and input RL, low IL in the passband region with high suppression level in the stopband. Moreover, the presented WPD provides a large attenuation of the second to seventh harmonics in the range of 2.54–13.48 GHz.
- Integration: the design and fabrication of multiport systems with improved performance characteristics are still a challenge for microwave researchers. In this paper, the presented LPF structure has been replaced in both branches of a conventional WPD as a means of improving harmonic suppression.
- Affordable: the size and cost of implementation are key factors to consider, so in this work, a key goal was to reduce circuit size to achieve a simple structure. A commercial high-frequency FR4 substrate with low cost and high accessibility has therefore been used in this work to ensure low cost of manufacture.
- Excellent results: according to Table 4, the WPD performance compares very well with, and improves on, the existing state-of-the-art. For instance, harmonic suppression for the second to seventh harmonics have been achieved with greater than 51.2, 40.3, 51.36, 37.5, 42.9 and 28.2 (all in dB) attenuation levels, respectively across a frequency range from 2.54 to 13.48 GHz.
- Applications: among power dividers, WPDs are widely used due to their simple construction, narrow bandwidth, and reliable performance. WPDs can be used in test systems to measure two different characteristics of a signal, such as frequency and power, for broadband-independent signal sampling. GSM modems have a wide range of applications in transaction terminals, supply chain management, and security applications. One of the common frequency bands for GSM is 1800 MHz and the proposed WPD shows good performance at this frequency, in terms of having a wide stop band, high isolation, and suppression of unwanted harmonics. In modern wireless communication systems, power dividers and filters are significant components used in non-linear circuits such as detectors^6^, power amplifiers^47,48^, mixers^49^, active circulators, and phase shifters^50^. Consequently, WPDs are designed to suppress unwanted harmonics that are generated based on non-linear electronics devices (harmonic balance theory) such as diodes and transistors. In this paper, the operating frequency is 1.8 GHz, and the proposed LPF structure can pass this frequency and suppress other harmonics. One such harmonic is the second harmonic (2*f*_0_ = 3.6 GHz) that lies in close proximity to the 5G New-Radio (NR) N-77 frequency band^51^, that should be suppressed for avoiding frequency interference. The presented WPD has been designed for operation at 1.8 GHz, so it can be used in next generation microwave circuits and state-of-the-art wireless communication systems for LTE and GSM services.

## Conclusion

This paper has presented a novel modified WPD with high isolation and high attenuation levels in the stopband. The desirable performance of the power divider is mainly attributed to a highly tailored LPF structure that has been incorporated in the WPD, removing harmonics up to the 7th harmonic and contributing to high isolation levels. The demonstrated results show an IL better than 3.52 dB at the operating frequency with an input RL and isolation greater than 34.2 dB and 31.2 dB, respectively. There is good agreement between the simulated and measured results. The proposed WPD is therefore suitable for use in a wide range of modern wireless communication systems in GSM and LTE services.

## Supplementary Information

Supplementary Information.

## Data Availability

The datasets used and/or analyzed during the current study are available from the corresponding author on reasonable request.
