# Supplementary material for: Design and fabrication of a Wilkinson power divider with harmonic suppression for LTE and GSM applications
Source: Sci Rep. 2023 Mar 14;13:4246. doi: 10.1038/s41598-023-31019-7 (PMC10015064; doi:10.1038/s41598-023-31019-7)
Supplement: Supplementary file 1 — Supplementary Information. [file 41598_2023_31019_MOESM1_ESM.pdf]

APPENDIX

Common Even and Odd-mode parameters:

$$Z_p = \frac{1}{2} Z_x = Z_x \,||\, Z_x = \frac{1}{2} Z_2 \frac{(-j\, Z_3 \cot\theta_3) + j\, Z_2 \tan\theta_2}{Z_2 + Z_3 \cot\theta_3 \tan\theta_2} \qquad (AP-1)$$

$$Z_m = Z_{m_1} \frac{(-j\, Z_{m_2} \cot\theta_{m_2}) + j\, Z_{m_1} \tan\theta_{m_1}}{Z_{m_1} + Z_{m_2} \cot\theta_{m_2} \tan\theta_{m_1}} \qquad (AP-2)$$

$$Z_R = Z_4 \frac{(-j\, Z_5 \cot\theta_5) + j\, Z_4 \tan\theta_4}{Z_4 + Z_5 \cot\theta_5 \tan\theta_4} \qquad (AP-3)$$

Even Mode:

$$Z_A = \frac{(-j\, Z_8 \cot\theta_8)\, Z_O}{-j\, Z_8 \cot\theta_8 + Z_O} \qquad (AP-4)$$

$$Z_B = Z_6 \frac{Z_A + j\, Z_6 \tan\theta_6}{Z_6 + j\, Z_A \tan\theta_6} \qquad (AP-5)$$

$$Z_C = Z_B \,||\, Z_p = \frac{Z_B\, Z_p}{Z_B + Z_p} \qquad (AP-6)$$

$$Z_D = Z_7 \frac{Z_C + j\, Z_7 \tan\theta_7}{Z_7 + j\, Z_C \tan\theta_7} \qquad (AP-7)$$

$$Z_E = Z_D \,||\, Z_m \,||\, Z_R = \frac{1}{\frac{1}{Z_D} + \frac{1}{Z_m} + \frac{1}{Z_R}} \qquad (AP-8)$$

$$Z_F = Z_7 \frac{Z_E + j\, Z_7 \tan\theta_7}{Z_7 + j\, Z_E \tan\theta_7} \qquad (AP-9)$$

$$Z_G = Z_F \,||\, Z_p = \frac{Z_F\, Z_p}{Z_F + Z_p} \qquad (AP-10)$$

$$Z_H = Z_6 \frac{Z_G + j\, Z_6 \tan\theta_6}{Z_6 + j\, Z_G \tan\theta_6} \qquad (AP-11)$$

$$Z_{in}^{even} = Z_1 \frac{Z_H + j\, Z_1 \tan\theta_1}{Z_1 + j\, Z_H \tan\theta_1} \qquad (AP-12)$$

Odd Mode:

$$Z_I = jZ_1 \tan\theta_1 \qquad (AP-13)$$

$$Z_J = Z_6 \frac{Z_I + j\, Z_6 \tan\theta_6}{Z_6 + j\, Z_I \tan\theta_6} \qquad (AP-14)$$

$$Z_K = Z_J \parallel Z_p = \frac{Z_J\, Z_p}{Z_J + Z_p} \qquad (AP-15)$$

$$Z_L = Z_7 \frac{Z_K + j\, Z_7 \tan\theta_7}{Z_7 + j\, Z_K \tan\theta_7} \qquad (AP-16)$$

$$Z_N = Z_L \parallel Z_m \parallel Z_R = \frac{1}{\frac{1}{Z_L} + \frac{1}{Z_m} + \frac{1}{Z_R}} \qquad (AP-17)$$

$$Z_Q = Z_7 \frac{Z_N + j\, Z_7 \tan\theta_7}{Z_7 + j\, Z_N \tan\theta_7} \qquad (AP-18)$$

$$Z_S = Z_Q \parallel Z_p = \frac{Z_Q\, Z_p}{Z_Q + Z_p} \qquad (AP-19)$$

$$Z_T = Z_6 \frac{Z_S + j\, Z_6 \tan\theta_6}{Z_6 + j\, Z_S \tan\theta_6} \qquad (AP-20)$$

$$Z_O = Z_8 \frac{{}^{R}\!/_{2} + j\, Z_8 \tan\theta_8}{Z_8 + j \frac{R}{2} \tan\theta_8} \qquad (AP-21)$$

$$Z_{in}^{odd} = Z_T \parallel Z_O = \frac{Z_T\, Z_O}{Z_T + Z_O} \qquad (AP-22)$$
